# On the Climate of California in Its Relation to the Treatment of Pulmonary Consumption

**Published:** 1860-09

**Authors:** James Blake

**Affiliations:** Sacramento


*On the Climate of California in its relation to the Treatment of Pulmonary Consumption.* By JAMES BLAKE, M. D., F. R. C. S.

The increasing importance that more correct views on the pathology and treatment of phthisis, is daily attaching to the subject of climate in its relation to this disease, has led me offer some few remarks on its treatment, in connection with the advantages that the climate of California offers, for carrying out the only rational means which, I believe, affords any hope of successfully treating the disease. An impression, founded on imperfect observation, has obtained, that there is something very prejudicial in our climate to consumptive patients. There can be no doubt but that a full proportion of our deaths are caused by phthisis, but I think this is to be attributed more to a large number of persons with a phthisical tendency, or even with the disease fully developed, having emigrated to our State in the hope of deriving benefit from the voyage and change of air, than to anything in the climate favorable to the development of tubercular disease. The habits of our population, so intensely devoted as they are to money-making, lead them to neglect the most common hygienic measures, and consequently tend to favor the development and fatal termination of the disease. And again, if phthisis is to be treated by expectorants, sedatives, tonics, and warm and *comfortable* rooms, our patient must die, even if the field of our practice had been in the garden of Eden. That pulmonary consumption, when once fully developed, can rarely, if ever, be cured by drugs, is a fact which experience is daily confirming, notwithstanding the specifics that are being constantly published by professed and medical quacks; but my faith in the curability of the disease is not at all diminished, although I have watched the failure of all these boasted specifics, from St. John Long's longissimusdorsi liniment to Dr. Churchill's hypophosphites. The more I have seen of the disease, the more I have been impressed with the impossibility of curing it with drugs, but at the same time, the more am I convinced that a large portion, even of those cases that have advanced to the second and third stages of the disease, are curable, and that there is not another spot in the civilized portions of the globe where their treatment can be undertaken with the same hope of success as in California. Such is the result of my own experience in the treatment of the disease in this country, and I shall be able to show that these facts, furnished by experience, are in accordance with the more correct views that have been advanced during the last few years, on its pathology. It is now generally admitted that pulmonary consumption is not a mere local disease

—that the deposition of tuberculous matter in the lungs is but the expression of a state of the system, brought about by the mal-assimilation of food, and although neither physiology nor pathology has succeeded in pointing out on what this mal-assimilation in individuals with a tuberculous tendency depends, yet the progress made towards a successful treatment of the disease by referring it back this one step—from the lungs to the digestive organs—is most important. If we now consider what are the principal indications to be followed in the treatment of a confirmed case of phthisis, according to our more correct pathological views, we shall have abundant proof of the advantages we possess in our climate of combating it with every prospect of success. The first great indication is to restore the functions of the digestive organs. To effect this, there is nothing to be compared to exercise and living in the open air. If the weather will permit, let the patient live in the open air; if he is not strong enough to walk, let him ride on horseback; if he cannot do this, let him go in a carriage; if he cannot bear exercise for more than ten minutes at a time, this is enough to begin on.—Avoid over-fatigue, but persevere; ten minutes' exercise every hour, or every two hours, will soon enable him to stand more; the more out-of-door exercise he can take, the more will the digestive powers become improved, and the more rapidly will strength be regained. During the summer months, if possible, let him camp out, sleep out under a tree, without tent or roof, save that of the spreading boughs. This is the most powerful means we possess for improving the digestive powers, and it is astonishing how well draught-fearing, cold-catching, coughing patients can stand it. I had patients sleeping out on the coast range, with unthawed snow still below them, during the inclement weather of our late extraordinarily cold June, without catching the slightest cold, without, as they informed me, so much as a sneeze, and with marked improvement in their general symptoms. The resistance to any ill-effects from cold and wet which can be obtained by living in the open air, would seem almost incredible to those who have not experienced it. Shortly after leaving England, in bad health, and at a time when I dared scarcely put my feet on a damp pavement without the certainty of an attack of catarrh, I made a trip beyond the western frontier of Texas, and had to pass more than one night drenched with rain, the water pouring under our blankets, and yet I experienced not the slightest ill effects. Those who have crossed the plains to this country can not but be aware how exposure to wet and cold can be safely endured when camping out. But this is merely a collateral, although a most important advantage to be attained in the out-door treatment of consumption. The most important point gained is the rapid improvement that living in the open air produces in the digestive functions. Few who have not gone through a course of open air for the cure of dyspepsia can realize it. Many instances have come under my observation, in which individuals, laboring under some form of irritable dyspepsia, yet living perhaps in favorable hygienic conditions, taking regular exercise, living on wholesome diet, and yet to whom a piece of fat bacon would be an abomination, and almost a poison; yet turn these dyspeptics out in the mountains, packing their blankets and provisions on their backs, and in the course of a few days they will be able to digest, and not only to digest, but to relish the fattest bacon that was ever made in Ohio; they will even be unwilling to lose the grease that drops from the luscious morsel as it broils before the camp fire, robbing the ashes of their perquisites by catching the hissing drops on their hard bread, which they then can eat with increased gusto. That these are facts I know by personal experience, and although they might appear to be trivial under a general point of view, yet they become invested with vital importance, when the state of thousands of our fellow-beings is dependent on their recognition and appreciation. What else, I would ask, is there that would produce such improvement in dyspeptic patients in so short a time, or even in weeks, or months? What have we amongst our bitters, tonics, or all the drugs in the pharmacopæa, that will begin to compare with out-door life in strengthening the digestive organs? and why is it that this powerful means of striking at the root of the disease, of producing that change in the condition of the system that alone can lead to a hope of ultimate recovery, has been so generally neglected? Admitting phthisis to be dependent on mal-assimilation, why has the profession, as a body, ignored a remedy which I believe to be as complete a specific in restoring the assimilative powers as is quinine in ague, or, at any rate, it is one that far surpasses every other. I am aware that consumptive patients are frequently sent to Madeira, to Havana, to the Southern States, and to the Sandwich Islands, for change of climate, and in some instances benfit has been derived from the change, more, however, I believe, from the amount of out-door life that has thus been incidentally enjoyed, than from there being anything in these climates directly favorable to removing the cause of the disease; on the contrary, I think all of them are inferior to California and Western Texas for consumptive patients, and some of them, particularly Havana and Southern Sates, are positively injurious, as far as regards the main objects to be aimed at—the strengthening of the digestive organs. The complete immunity of our climate from rain during so many months of the year, the dry, bracing air of our mountains, so completely free from every taint of malaria, affords us facilities for carrying out the open air treatment of phthisis, which are to be found in few places in the civilized portions of the globe, particularly in the temperate zone; and those who regard California as unfavorable for consumptive patients, must have entirely ignored the importance of living in the open air as an element in curing the disease. As a general rule, patients may live in the open air with perfect safety from the middle of October; or, if some protection from rain should be required during that period, it certainly would have to be used but during a few days. During the winter months patients would have to avail themselves of houses; but even then a large portion of their time can be passed in the open air, as there are few temperate climates where, even during the rainy season, there are so many clear days as in California.

I must reserve, until I allude to the pharmaceutical treatment of the disease, any remarks on the medicines that may be required in some cases for strengthing the digestive organs, and will now shortly point out the advantages afforded by the open air treatment in allaying or moderating some of the other symptoms that are so frequently met with in cases of phthisis. I have already alluded to the increased power of resistance to atmosphere changes, which is attained by living in the open air; this is an important advantage, as it preserves our patients, in a great measure, from those intercurrent attacks of sub-aceut pneumonia, which so often favor the development of tubercle.—The irritability of the bronchial and laryngeal mucous membrane becomes diminished, so that the cough is reduced in frequency, until it is no more than is required for clearing the lungs of their secretions. That this can be effected without nauseating expectorants, or anti-digestive sedatives, is a great gain, as our patient thus secure rest for their sore lungs, and good sleep without thier stomachs being interfered with. The excessive nervous irritability that, independently of cough, frequently interfered with sleeping, is more effectually allayed by living in the open air than by any other means; the soothing influence of exercise in the open air on an airritable nervous system, is an uni_versally acknowledged fact, and of all sedatives, it is that which is most appropriate in cases of consumption. The night sweats, as a general rule, are checked by sleeping in the open air, or in thoroughly ventilated rooms; although this is a symptom against which we are frequently obliged to administer medicines, fortuneately they can be selected from amongst substances that rarely interfere with the digestive functions. I must reserve for a future article a short synopsis of the pharmaceutical treatment of the disease, and also the details of the manner in which the most important element of our treatment, the hygienic, can be carried out. It is often difficult to induce the more civilized class of our patients to fall back to the nomadic state. Many cannot do it; their social position will not allow it. Many will not from fear that they must die if they get five miles from an apothecary shop. Obstacles will present themselves which cannot be overcome, and which a compromise must be effected. As my object is to point out how phthisis can be cured, and not merely the principles od which it is curable, I will endeavoa, in a subsequent article, to show how these compromises can be best carried out, so as to give every class of our patients a fair chance of recovery. I shall also review the subject of the varieties of climate to be found in our State, and of the best localities for our patients at different seasons of the year.—*Pacific Medical & Surgical Journal.*

Sacramento, July, 13th, 1860.

